# A Photochemoenzymatic Hunsdiecker‐Borodin‐Type Halodecarboxylation of Ferulic Acid

**DOI:** 10.1002/cbic.202200367

**Published:** 2022-08-23

**Authors:** Claudio Zippilli, Miguel Jimenez Bartolome, Thomas Hilberath, Lorenzo Botta, Frank Hollmann, Raffaele Saladino

**Affiliations:** ^1^ Department of Biological and Ecological Sciences University of Tuscia Via S.C. De Lellis s.n.c. 01100 Viterbo Italy; ^2^ Department of Biotechnology Delft University of Technology Van der Maasweg 9 Delft The Netherlands; ^3^ Institute of Environmental Biotechnology University of Natural Resources and Life Sciences, Vienna Konrad Lorenz Strasse 20 3430 Tulln Austria

**Keywords:** halodecarboxylation, phase-transfer catalysis, photobiocatalysis, photochemoenzymatic reactions, vanadium chloroperoxidase

## Abstract

A photochemoenzymatic halodecarboxylation of ferulic acid was achieved using vanadate‐dependent chloroperoxidase as (bio)catalyst and oxygen and organic solvent as sole stoichiometric reagents in a biphasic system. Performance and selectivity were improved through a phase transfer catalyst, reaching a turnover number of 660.000 for the enzyme.

## Graphical abstract

**Halodecarboxylation** of α,β‐unsaturated ferulic acid was achieved by combining photo‐, bio‐ and phase transfer catalysis in a biphasic system. Reactive hypobromite was formed *in situ* by combining Blue‐LED and vanadium chloroperoxidase, and efficiently transferred from the aqueous to the organic layer by a phase‐transfer catalyst, improving overall performance and selectivity.

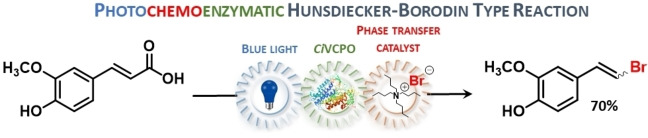

## Introduction

Lignin is the second most abundant natural polymer accounting for about 30 % of the organic carbon in the biosphere, representing an attractive starting material for the synthesis of biobased value‐added compounds.[^1^, ^2^] Interestingly, past efforts have largely focused on the depolymerisation of lignin while the utilisation of lignin‐derived monomers have so far been far less in focus. Lignin is rich in α,β‐unsaturated carboxylic acids such as ferulic acid (**1**) making it an attractive target for further transformations.^[3]^ In this context, the Hunsdiecker‐Borodin reaction, also known as halodecarboxylation, is a versatile method to access vinyl halides as multi‐functional building blocks.[^4^, ^5^]

Despite decades of intensive research, Hunsdiecker‐Borodin type reactions still suffer from some unresolved issued such as often requiring multistep processes, heavy metal catalysts, highly toxic and corrosive electrophilic halide sources, and halogenated solvents.[^6^, ^7^, ^8^]

Currently N‐halo succinimides (NXS) are widely used as less environmentally demanding and easier to handle sources of electrophilic halides.[^4^, ^9^, ^10^] Nevertheless, NXSs are used in stoichiometric amounts resulting in significant wastes that not only negatively impact the atom efficiency of these reactions but also complicate product isolation.

In this context, enzymatic *in situ* generation of electrophilic halide species using haloperoxidases may represent an interesting catalytic alternative to NXS.[^11^, ^12^, ^13^]

Haloperoxidases catalyse the H_2_O_2_‐dependent oxidation of halides into the corresponding hypohalites. The latter then electrophilically attack the C=C‐double bond of the α,β‐unsaturated carboxylic acid starting material yielding the vinyl halides in an overall chemoenzymatic reaction.

## Results and Discussion

**Scheme 1:**
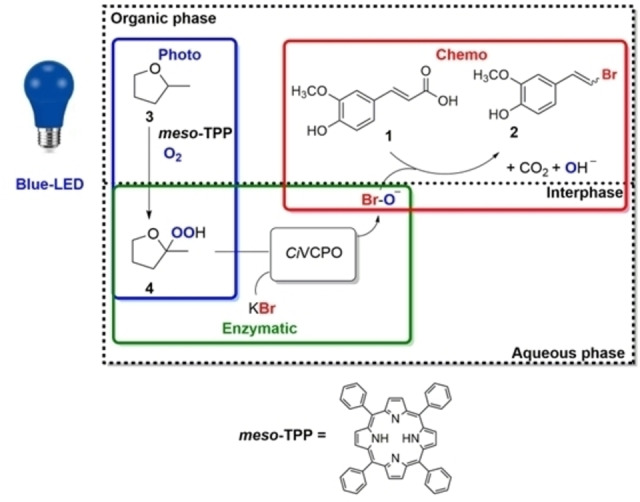
Envisioned photochemoenzymatic cascade comprising photocatalytic (*meso*‐TPP mediated) oxygenation of 2‐methyl‐THF **3** to 2‐hydroperoxo‐2‐methyl‐THF **4**; the latter serves as stoichiometric oxidant for the enzymatic oxidation of bromide to hypobromite which then initiates the brominative dehalogenation of ferulic acid **1** to **2**.

For our investigations we used the vanadate‐dependent chloroperoxidase from the fungus *Curvularia inaequalis* (*Ci*VCPO).[^14^, ^15^, ^16^] Ferulic acid **1** served as model compound. For the photocatalytic *in situ* generation of organic hydroperoxides to drive the enzymatic hypohalite generation we relied on the previously reported *meso*‐tetraphenylporphyrin (*meso*‐TPP) photocatalyst to mediate the photocatalytic hydroperoxygenation of 2‐methyl tetrahydrofuran **3** into the corresponding 2‐hydroperoxide **4** (Scheme 1).^[17]^

The system relies on the tetrapyrrolic photosensitizer *meso*‐TPP (Scheme 1) that, once excited by blue light (460–470 nm) to its triplet state, can transfer excitation energy to oxygen molecules bringing them into their reactive singlet excited state.^[18]^ ^1^O_2_ selectively reacts with the α‐ethereal carbon atom of 2‐MeTHF **3** leading to the corresponding hydroperoxide **4** ^[19]^ that serves as stoichiometric oxidant for the enzymatic oxidation of bromide to hypobromite.

Illuminating a biphasic reaction system (2‐Me‐THF and aqueous buffer, 1 : 1 v/v) containing 2.0 mM_org_ ferulic acid, 0.4 mM_org_ *meso*‐TPP (20 mol‐%), 2.0 mM_aq_ (1 eq) KBr and 1.0 μM_aq_ *Ci*VCPO (0.05 mol‐%) with blue LED light for 72 h resulted in 50 % conversion of the initial starting material at approx. 50 % selectivity (Table 1, entry 1).

**Table 1:** Influence of some reaction parameters on the result of the proposed photochemoenzymatic Hunsdiecker‐Borodin type decarboxylative halogenation of ferulic acid.

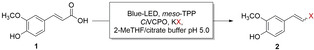						
Entry^[a]^	[KX] [mM]	X	Time [h]	Conversion [%]^[b]^	Yield [%]^[c]^	*E/Z*
1	2.0	Br	72	50	28	*90 : 10*
2^[d]^	0	–	72	>99	–	*n.d*.
3^[e]^	2.0	Br	72	–	–	*n.d*.
4^[f]^	2.0	Br	72	–	–	*n.d*.
5	2.0	Br	96	95	32	*85 : 15*
6	2.0	Br	120	98	28	*70 : 30*
7	3.0	Br	96	94	42	*85 : 15*
8	4.0	Br	96	98	31	*85 : 15*
9	5.0	Br	96	98	16	*85 : 15*
10	3.0	Cl	96	91	37	*85 : 15*

[a] Reaction conditions: 2.0 mM ferulic acid and 400 μM *meso*‐TPP in 500 μL of 2‐MeTHF; 1.0 μM *Ci*VCPO and 2.0, 3.0, 4.0 or 5.0 mM KX (X= Br; Cl) in 500 μL of citrate buffer pH 5.0, 0.1 M for 72, 96 or 120 hours. [b] Conversion determined by HPLC. [c] Yield determined by HPLC. [d] Reaction without KBr. [e] Reaction without *Ci*VCPO. [f] Reaction with thermally inactivated *Ci*VCPO. n.d.=not determined. Reactions were conducted in technical duplicates.

The poor selectivity of the reaction can be assigned to a direct, yet unidentified reaction of *Ci*VCPO with the starting material as reactions in the absence of a bromide source gave full conversion of the starting material but (expectedly) no product formation (Table 1, entry 2). Performing the experiments in the absence of enzyme or using thermally inactivated *Ci*VCPO (Table 1, entries 3, 4) resulted in complete recovery of the starting material.

Quite interestingly, increasing the concentration of the halide (Table 1, entries 6–9) also decreased the selectivity (Product yield/ substrate conversion) of the overall reaction. Prolonging the reaction time had no significant effect on the selectivity but rather on the product distribution (E/Z ratio), which most likely can be rationalised by photochemical double bond isomerisation.^[20]^

Exchanging potassium bromide by chloride under so far optimised reaction conditions in terms of yield (Table 1, entry 7) lead to vinyl chloride in comparable yield (Table 1, entry 10).

**Figure 1:**
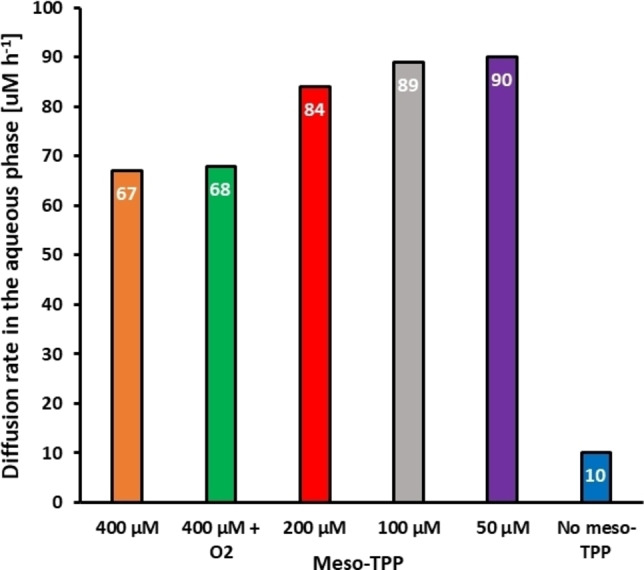
Effect of molecular oxygen and [*meso*‐TPP] on the photocatalytic hydroperoxidation of **3** to **4**. Extrapolated diffusion rate of **4**. [*meso*‐TPP]=400 μM (orange), 400 μM with addition of molecular oxygen (green), 200 μM (red), 100 μM (grey), 50 μM (purple), 0 μM (control, blue). Reactions were conducted in technical duplicates.

Next, we investigated the influence of O_2_‐concentration as well as concentration of *meso*‐TPP on the accumulation of the hydroperoxide in the aqueous layer (Figure 1, Figure S3). Changing the gaseous phase from ambient air (c(O_2_) ca. 9.3 mM to pure O_2_ (c(O_2_)=44.6 mM) did not influence the hydroperoxide accumulation rate in the aqueous layer. Interestingly however, decreasing the photocatalyst concentration positively influenced the hydroperoxide accumulation. Possibly, non‐productive cross‐relaxation (self‐quenching) of *meso*‐TPP accounts for this observation. The hydroperoxide formation rate in the absence of *meso*‐TPP was marginal.

In all tested conditions compound **4** reached an equimolar concentration with respect to substrate **1** after 48 hours (Figure S3), not explaining the time latency of the reaction (96 h).

As also these experiments did not result in a more in‐depth understanding of the overall rate‐limiting factor of the overall reaction, we determined the partitioning of the starting material (ferulic acid) between the organic and aqueous layer (Figure S17). Rather surprisingly, we found that more than 95 % of the initial ferulic acid resided in the organic layer (pK_a_ (ferulic acid)=4.6 while the pH of the aqueous layer was 5). Therefore, we reasoned that the reaction between aqueous‐borne HOBr and ferulic acid (residing in the 2‐Me‐THF phase) may be overall rate‐limiting. Therefore, we applied tetrabutylammonium bromide (TBAB) as phase transfer catalyst (PTC) and Br‐source to increase the availability of OBr‐ in the organic layer (Table 2).^[21]^

**Table 2:** Influence of a phase transfer catalyst and enzyme concentration on the performance of the photochemoenzymatic Hunsdiecker‐Borodin type decarboxylative halogenation of ferulic acid.

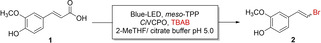					
Entry^[a]^	[*Ci*VCPO] [nM]	[TBAB] [mM]	Conversion [%]^[b]^	Yield [%]^[c]^	*E/Z*
1	1000	3.0	78	51	*80 : 20*
2	1000	4.0	>99	71	*85 : 15*
3	1000	5.0	>99	60	*n.d*.
4	1000	6.0	>99	55	*n.d*.
5	10	4.0	>99	70	*85 : 15*
6	1	4.0	35	33	*85 : 15*
7	0.1	4.0	3	2	*n.d*.

[a] Reaction conditions: 2.0 mM ferulic acid and 50 μM *meso*‐TPP in 500 μL of 2‐Me‐THF; 0.1 nM‐1.0 μM *Ci*VCPO and, 3.0, 4.0, 5.0 or 6.0 mM TBAB in 500 μL of citrate buffer pH 5.0, 0.1 M for 48 hours. [b] Conversion determined by HPLC. [c] Yield determined by HPLC. n.d.=not determined. Reactions were performed in independent duplicates.

Gratifyingly, applying 3.0 mM (1.5 eq) of TBAB lead not only to a very significant acceleration of the reaction but also improved the selectivity (Figure S11). This was improved even further in the presence of 4.0 mM (2.0 eq) of TBAB yielding HPLC‐grade product in the organic layer (Figure S13). Further increasing the TBAB concentration decreased the selectivity (Table 2, entries 2–4). The last observation most likely can be assigned to further ring‐halogenation of the desired vinyl bromide product (Figure S9).

Next, we aimed at investigating the influence of the biocatalyst concentration on the overall performance (Table 2, entries 5–7). Only upon reduction of the *Ci*VCPO concentration to 1.0 nM, 0.00005 mol‐%) we observed a reduction of the overall conversion (albeit concomitant with a significant improvement in the selectivity!). From this experiment a total turnover number (TONs=mol_product_⋅mol_enzyme_^–1^) of approx. 660.000 for *Ci*VCPO corresponding to an average turnover frequency (TOF=TONs ⋅ s^−1^) of 3.8 s^−1^ (over 48 h) can be estimated, which is in line with some previously determined catalytic performances of the biocatalyst.[^11^, ^12^]

**Scheme 2:**
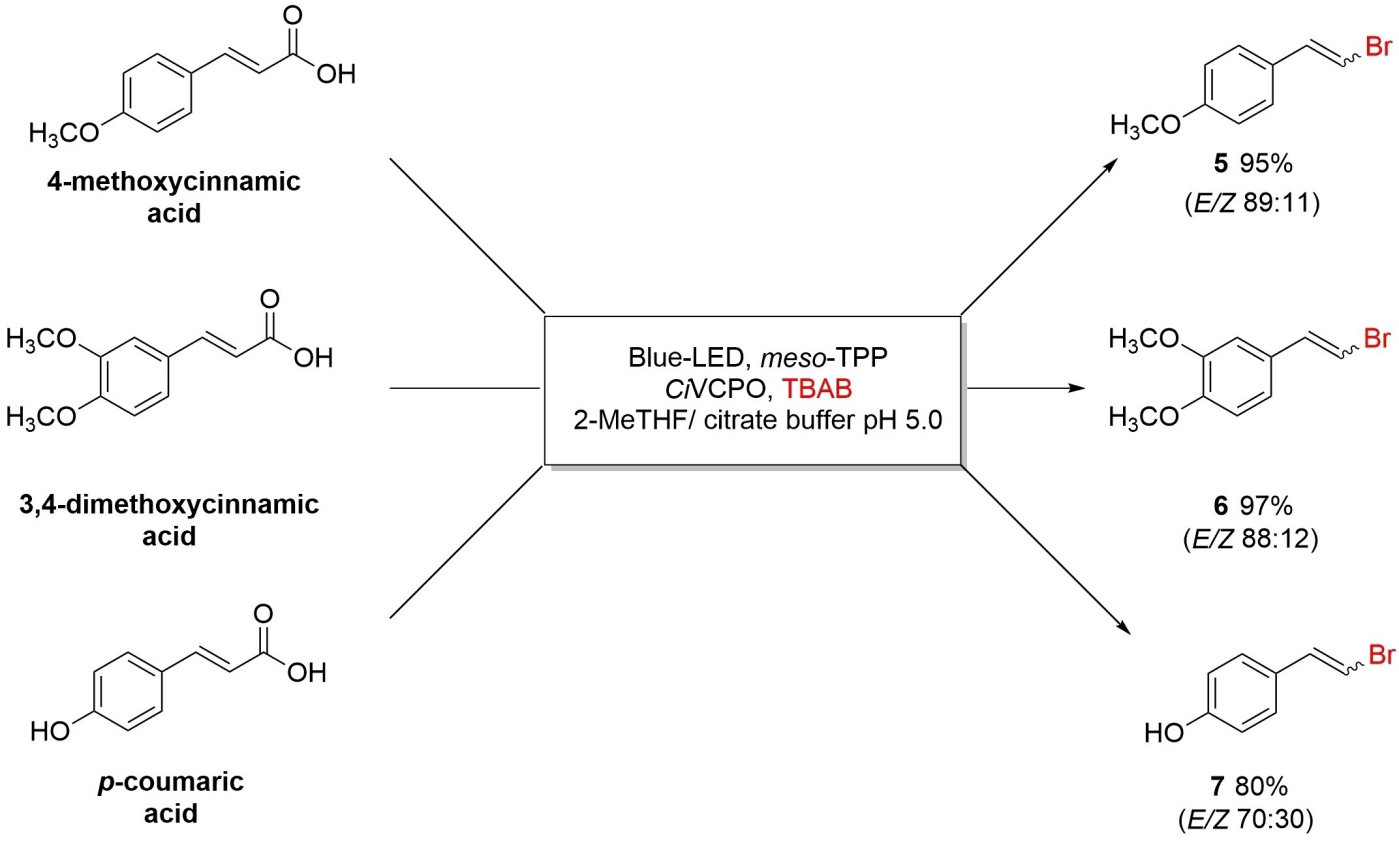
Photochemoenzymatic Hunsdiecker‐Borodin type reaction on 4‐methoxycinnamic, 3,4‐dimethoxycinnamic and *p*‐coumaric acid. Reaction conditions: 2.0 mM substrate and 50 μM *meso*‐TPP in 500 μL of 2‐Me‐THF; 10.0 nM *Ci*VCPO and 4.0 mM TBAB in 500 μL of citrate buffer pH 5.0, 0.1 M for 48 hours. Yields were determined by HPLC. Reactions were performed in duplicates.

The photochemoenzymatic halodecarboxylation was then extended to lignin‐derived 4‐methoxycinnamic, 3,4‐dimethoxycinnamic and *p*‐coumaric acids, obtaining corresponding vinyl bromide **5**, **6** and **7** in 95, 97 and 80 %, respectively (Scheme 2, Figure S14‐16).

**Figure 2:**
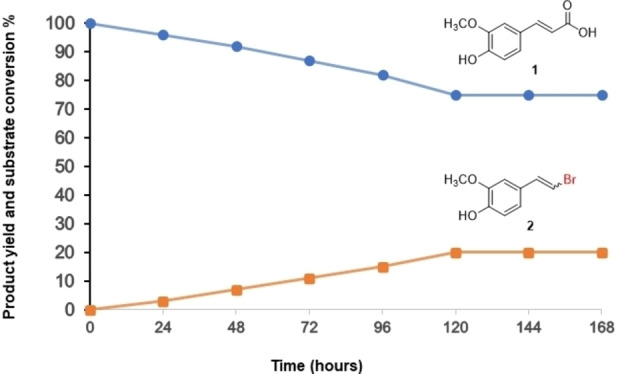
Time course of product yield (orange) and substrate conversion (blue) of the semi‐preparative scale photochemoenzymatic Hunsdiecker‐Borodin type reaction on ferulic acid **1**. Reaction conditions : 30.0 mM ferulic acid and 50 μM *meso*‐TPP in 5.0 mL of 2‐Me‐THF; 10.0 nM *Ci*VCPO, 4.0 mM TBAB and 56.0 mM KBr in 5.0 mL of citrate buffer pH 5.0, 0.2 M for 168 hours.

We finally attempted to scale up the photochemoenzymatic process on a semi‐preparative scale with ferulic acid **1** as selected substrate. We relied on the so far optimised reaction conditions (Table 2, entry 5) but increasing substrate concentration from 2.0 to 30.0 mM and offsetting the bromide source (TBAB 4.0 mM) with potassium bromide (56.0 mM). Stirring the reaction under blue‐LED irradiation for 168 hours (7 days) an overall yield of 20 % of **2** has been obtained with a linear accumulation of vinyl bromide during five days (Figure 2). After this period, substrate conversion stopped abruptly. Currently, we are lacking a satisfactory explanation for the low robustness of the reaction. Possibly, photobleaching of *meso*‐TPP may account for this; but further in‐depth investigations will be necessary to clarify and debottleneck this issue.

## Conclusion

Overall, with this study we have demonstrated that photochemoenzymatic Hunsdieker‐Borodin‐type halodecarboxylation reactions are feasible paving the way to reactions utilising ambient air and 2‐Me‐THF as sole stoichiometric reagents. Admittedly, this reaction concept is still in its infancy. Especially increasing the activity (reaching preparative attractive space‐time yields) and robustness of the proposed photochemoenzymatic reaction scheme will be in focus of future studies. Particularly, modified photocatalysts exhibiting tailored activity and robustness may open up new synthetic possibilities.^[22]^

Nevertheless, we are convinced to have provided a promising starting point for these investigations.

## Experimental Section

**Optimal conditions for the photochemoenzymatic Hunsdiecker‐Borodin type reaction in analytical scale**: In a 4.0 mL glass vial, 500 μL of 2‐methyltetrahydrofuran containing α,β‐unsaturated carboxylic acid (2.0 mM) and *meso*‐TPP (50 μM) was combined with 500 μL of citrate buffer pH 5.0, 0.1 M, containing *Ci*VCPO (10 nM) and TBAB (4.0 mM). The reaction was gently stirred at 200 rpm at room temperature for 48 hours in a jacketed beaker with commercial blue‐LEDs (24 W) wrapped around. The reaction was stopped by adding 1.0 mL of ethyl acetate. The organic layer was then separated from the aqueous one and the latter extracted four times with ethyl acetate (4×1.0 mL). Organic fractions were combined, and the solvent evaporated under vacuum. The so obtained crude mixture has been analysed by HPLC.

**Reaction conditions for the photochemoenzymatic Hunsdiecker‐Borodin type reaction on semi‐preparative scale**: In a 20.0 mL glass vial, 5.0 mL of 2‐methyltetrahydrofuran containing ferulic acid (30.0 mM) and *meso*‐TPP (50 μM) was combined with 5.0 mL of citrate buffer pH 5.0, 0.2 M, containing *Ci*VCPO (10 nM), TBAB (4.0 mM) and KBr (56.0 mM) The reaction was gently stirred at 200 rpm at room temperature for 168 hours (7 days) in a jacketed beaker with commercial blue‐LEDs (24 W) wrapped around. The reaction was stopped by adding 10.0 mL of ethyl acetate. The organic layer was then separated from the aqueous one and the latter extracted four times with ethyl acetate (4×5.0 mL). The organic fractions were combined, washed with BRINE (1×30.0 mL), dried over sodium sulfate and evaporated under vacuum. The obtained crude mixture was purified by flash column chromatography using ethyl acetate/petroleum ether (1 : 7) as mobile phase, obtaining vinyl bromide **2** in 20 % isolated yield.

## Conflict of interest

The authors declare no conflict of interest.

1

## Supporting information

As a service to our authors and readers, this journal provides supporting information supplied by the authors. Such materials are peer reviewed and may be re‐organized for online delivery, but are not copy‐edited or typeset. Technical support issues arising from supporting information (other than missing files) should be addressed to the authors.

Supporting Information

## Data Availability

The data that support the findings of this study are available in the supplementary material of this article.
