# Supplementary material for: A Photochemoenzymatic Hunsdiecker‐Borodin‐Type Halodecarboxylation of Ferulic Acid
Source: Chembiochem. 2022 Aug 23;23(19):e202200367. doi: 10.1002/cbic.202200367 (PMC9804872; doi:10.1002/cbic.202200367)
Supplement: Supplementary file 1 — Supporting Information [file CBIC-23-0-s001.pdf]

ChemBioChem

Supporting Information

A Photochemoenzymatic Hunsdiecker-Borodin-Type Halodecarboxylation of Ferulic Acid

Claudio Zippilli, Miguel Jimenez Bartolome, Thomas Hilberath, Lorenzo Botta,*
Frank Hollmann,* and Raffaele Saladino

Table of contents

1. ***Materials***
2. ***Production of CiVCPO*** *(Figure S1)*
3. ***Analytical procedure***
4. ***Chemical synthesis and characterization of product standards 2 and 5-7***
5. ***Determination of the hydroperoxide 4 concentration*** *(Figure S2-4)*
6. ***Reaction conditions for the photochemoenzymatic Hunsdiecker-Borodin type reaction***
   - *6.1 HPLC chromatograms of experiments in Table 1 (Figure S5-8)*
   - *6.2 HPLC chromatograms of experiments in Table 2 (Figure S9-11)*
   - *6.3 Comparison of chemically and photochemoenzymatically obtained product* ***2*** *and HPLC purity grade (Figure S12,13)*
   - *6.4 Comparison of chemically and photochemoenzymatically obtained product* ***5***,***6*** *and* ***7*** *(Figure S14-16)*
7. ***Partition coefficient of ferulic acid in the biphasic system under different TBAB concentrations*** *(Figure S17-18)*
8. ***^{1}H-NMR of product 2 isolated from the semi-preparative photochemoenzymatic reaction.***

1. Materials

All reagents, solvents and standards were purchased form Sigma-Aldrich in the highest purity available and used without further purification except for 2-methyltetraidrofuran that was freshly distilled, degassed and stored under Argon prior to use it. All stock solutions used in this study were prepared with freshly distilled and degassed 2-methyltetrahydrofuran, stored under argon atmosphere in amber glass bottles to avoid any autoxidation processes.

2. Production of *Ci*VCPO

*Ci*VCPO was produced in recombinant *E. coli* TOP10 pBADgIIIB VCPO following previously described procedures.[1]

*Ci*VCPO was purified via heat treatment according to a modified protocol from literature.[1] 33 g of *E. coli* TOP10 pBADgIIIB VCPO were resuspended in around 100 mL 20 mM Tris-H_2SO_4 buffer (pH 8.1) containing 0.1 mM sodium-orthovanadate. One protease inhibitor tablet (cOmplete ULTRA Tablets, Mini, EDTA-free, Roche) and bovine DNAse I (Merck) with a spatula tip of $MgSO_4$ were further added prior to cell disruption. Cells were disrupted by use of a Multi Shot Cell Disruption System (3 cycles) at 1.5 kBar. The soluble fraction (crude cell extract) was separated from the cell debris by centrifugation (47.850 x *g* for 30 min at 4°C) and subsequently exposed at 70°C for 30 min. Then, heat precipitated proteins were removed by centrifugation (47.850 x *g* for 30 min, at 4 °C). The supernatant containing *Ci*VCPO was concentrated to around 20 mL (Amicon filters with 30 kDa cut-off membrane) and washed four times with fresh 20 mM Tris-H_2SO_4 buffer (pH 8.1) containing 0.1 mM sodium-orthovanadate. The final VCPO fraction (20 mL) was flash frozen in a roundbottom flask with liquid nitrogen and lyophilized at 0.1 mbar and -58 °C. Around 200 mg lyophilized *Ci*VCPO with a purity of 87% (according to SDS-PAGE analysis, see Figure S1) and an enzyme concentration of 5 nmol per mg lyophilized powder was obtained.

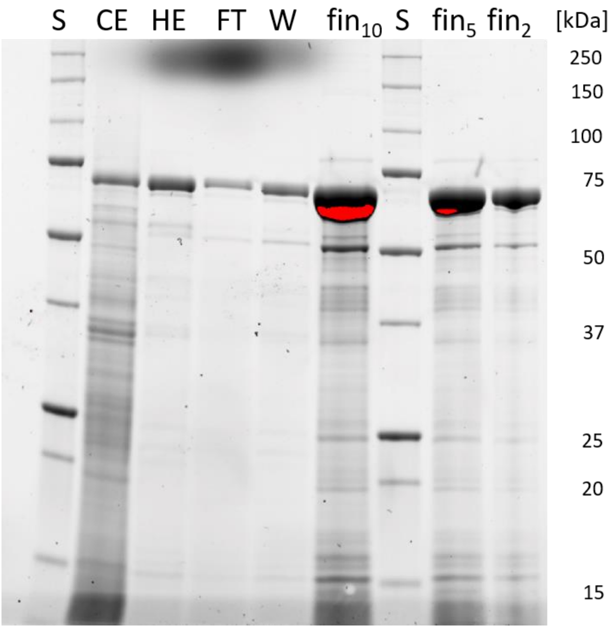

Figure S1: SDS-PAGE analysis following the purification of *Ci*VCPO. CE: crude extract, HE: soluble fraction after heat treatment, FT: flow through during concentration with Amicon filter (30kDa cutoff), W: Wash fraction with buffer during concentration with the Amicon, fin: final fraction used for lyophilisation. 2, 5 or 10 µg protein of each sample was applied (indicated by the indices). A purity of 87% VCPO was determined using the band intensities (5 µg protein load). S: Precision Plus Protein All Blue Standard (Biorad).

3. Analytical procedure.

HPLC measurements were performed using an Ultimate 3000 Rapid Resolution UHPLC system (ThermoFisher scientific) equipped with Alltima C18 (250 mm×4.6 mm, 5 µm) column and a multi-wavelength detector using the following method:

Time	Flow	H_2O 0.1% TFA	Acetonitrile
0	1.0	90	10
3.0	1.0	45	55
8.0	1.0	45	55
9.0	1.0	20	80
14.0	1.0	20	80
15.0	1.0	1	99
18.0	1.0	1	99
18.5	1.0	90	10
21.0	1.0	90	10

All measurements have been performed at least as duplicates from independent experiments. Conversions and yields are always based on calibration curves with authentic substrate standards and with chemically synthetized product standards.

Compound	Retention Time (min)
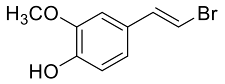 **2** (*E*)	8.83
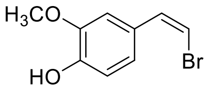 **2** (*Z*)	8.35
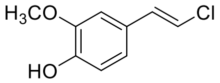 **2b** (*E*)	8.79
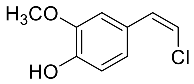 **2b** (*Z*)	8.27
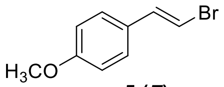 **5** (*E*)	12.43
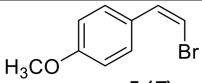 **5** (*Z*)	12.15
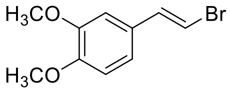 **6** (*E*)	11.35
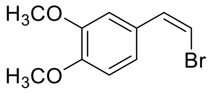 **6** (*Z*)	10.71
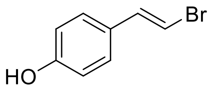 **7** (*E*)	8.63
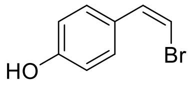 **7** (*Z*)	8.09

4. **Chemical synthesis and characterization of product standards 2 and 5-7**

Materials

All reagents and solvents were purchased form Sigma-Aldrich in the highest purity available and used without further purification. Reactions were carried out under argon atmosphere using standard Schlenk techniques. Reaction was monitored using thin layer chromatography on precoated aluminum silica gel Merck 60 F254 plates and a UV lamp (λmax=254 nm) was used for visualization. Merck silica gel 60 (230–400 mesh) was used for flash chromatography applying the indicated mobile phase. Product was dried in high vacuum (10-3 mbar). ^{1}H NMR and ^{13}C NMR were recorded on a Bruker Avance DRX400 (400 MHz/100 MHz) spectrometer using CD_3OD as solvent. Chemical shifts for ^{1}H and ^{13}C spectra were recorded in parts per million (ppm) on the δ scale. Coupling constants (J) are reported in Hz. Multiplicities are reported in the conventional form: s=singlet, d=doublet, t=triplet, td=triplet of doublets, q=quartet, m= multiplet, br s=broad singlet. The purity of product standards has been quantified by NMR using ethylene carbonate as internal standard and then used as standard for HPLC calibration curve of products.

Procedure

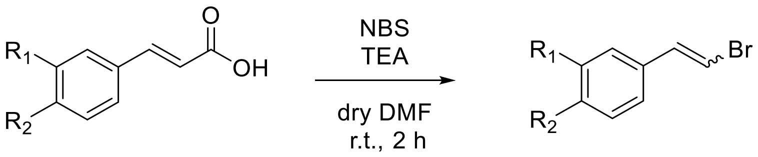

The selected α,β-unsaturated carboxylic acid (1.0 mmol, 1.0 eq.) and triethylamine (TEA) (5.0 mol%) were dissolved in 3.0 mL of dry *N*,*N*-dimethylformamide (DMF) under inert atmosphere (argon). The resulting solution was stirred for 5 minutes at room temperature then, *N*-Bromosuccinimide (1.05 mmol, 1.05 eq.) was added, and the reaction was further stirred for 2 hrs at room temperature. After this time, 20.0 mL of ethyl acetate (AcOEt) were added to the reaction and the resulting organic layer was washed with LiCl 3% aqueous solution (3 x 30 mL), distilled water (1x 30 mL) and BRINE (1x30 mL), dried over sodium sulfate and evaporated under vacuum. The obtained crude was purified by flash column chromatography using the indicated mobile phase.

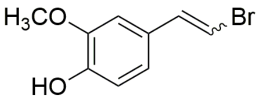

2 (*E/Z 95:5*)

***(E)*-Vinyl bromide 2.** Rf=0.23 (AcOEt/P.E. 1:2); white solid (25%). NMR purity 95%. ^{1}H-NMR (400 MHz, CD_3OD): δ= 7.04 (d, 1H, *J*= 14.0 Hz), 6.97 (d, 1H, *J*= 1.8 Hz), 6.83 (dd, 1H, *J*= 4.0, 8.0 Hz), 6.82 (d, 1H, *J*= 14.0 Hz), 6.76 (d, 1H, *J*= 8.4 Hz), 3.87 (s, 3H) ppm; ^{13}C-NMR (100 MHz, CD_3OD): δ= 147.2, 146.2, 136.3, 127.5, 118.9, 114.3, 108.2, 102.3, 54.4 ppm.

***(Z)*-Vinyl bromide 2.** The Z-form of vinyl bromide has been identified by characteristic signals at 6.35 (d, 1H, *J*= 8.0 Hz), and 3.88 (s, 3H) ppm.

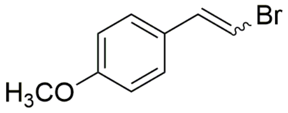

5 (E/Z 94:6)

(*E*)-Vinyl bromide 5. Rf=0.21 (AcOEt/P.E. 1:60); pale yellow oil (30%). ^{1}H-NMR (400 MHz, CD_3OD): δ= 7.32 (d, 2H, *J*= 8.8 Hz), 7.08 (d, 1H, *J*= 14.0 Hz), 6.90 (d, 2H, *J*= 8.8 Hz), 6.82 (d, 1H, *J*= 14.0 Hz), 3.80 (s, 3H) ppm; ^{13}C-NMR (100 MHz, CD_3OD): δ= 159.2, 135.0, 128.1, 126.5, 113.2, 102.8, 53.7 ppm.

***(Z)*-Vinyl bromide 5.** The Z-form of vinyl bromide has been identified by characteristic signals at 6.39 (d, 1H, *J*= 8.0 Hz), and 3.82 (s, 3H) ppm.

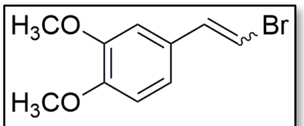

6 (*E/Z* 91:9)

***(E)*-Vinyl bromide 6.** Rf=0.18 (AcOEt/P.E. 1:5); pale yellow solid (35%). ^{1}H-NMR (400 MHz, CD_3OD): δ= 7.28 (d, 1H, *J*= 8.4 Hz), 7.27 (d, 1H, *J*= 8.4 Hz), 7.04 (d, 1H, *J*= 14.0 Hz), 6.90 (s, 1H), 6.88 (d, 1H, *J*= 13.6 Hz), 3.84 (s, 3H), 3.83 (s, 3H) ppm; ^{13}C-NMR (100 MHz, CD_3OD): δ= 152.8, 152.6, 140.0, 132.6, 122.7, 114.8, 112.3, 107.2, 58.4, 53.3 ppm.

***(Z)*-Vinyl bromide 6.** The Z-form of vinyl bromide has been identified by characteristic signals at 6.41 (d, 1H, *J*= 8.0 Hz), and 3.86 (s, 3H) ppm.

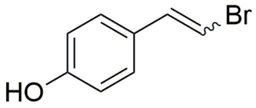

7 (E/Z 98:2)

***(E)*-Vinyl bromide 7.** Rf=0.18 (AcOEt/P.E. 1:5); pale yellow solid (20%). ^{1}H-NMR (400 MHz, CD_3OD): δ= 7.22 (d, 2H, *J*= 8.4 Hz), 7.04 (d, 1H, *J*= 13.6 Hz), 6.765 (d, 2H, *J*= 14.0 Hz), 6.760 (d, 1H, *J*= 8.8 Hz), ppm; ^{13}C-NMR (100 MHz, CD_3OD): δ= 156.9, 136.1, 127.0, 126.5, 114.5, 101.9 ppm.

***(Z)*-Vinyl bromide 7.** The Z-form of vinyl bromide has been identified by characteristic signals at 6.33 (d, 1H, *J*= 8.8 Hz) ppm.

^{1}H-NMR of *E/Z* (95:5) mixture of standard product 2

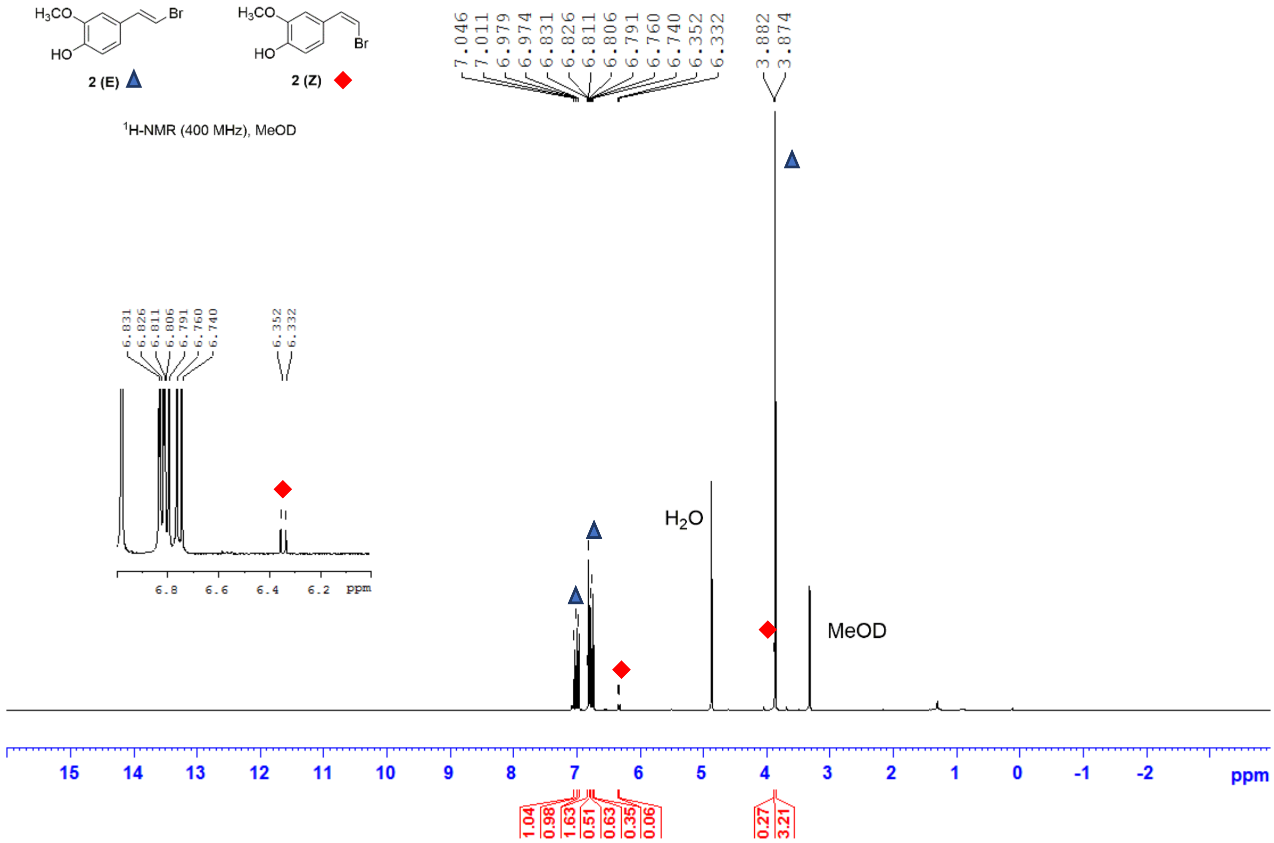

^{13}C-NMR of *E/Z* (95:5) mixture of standard product 2

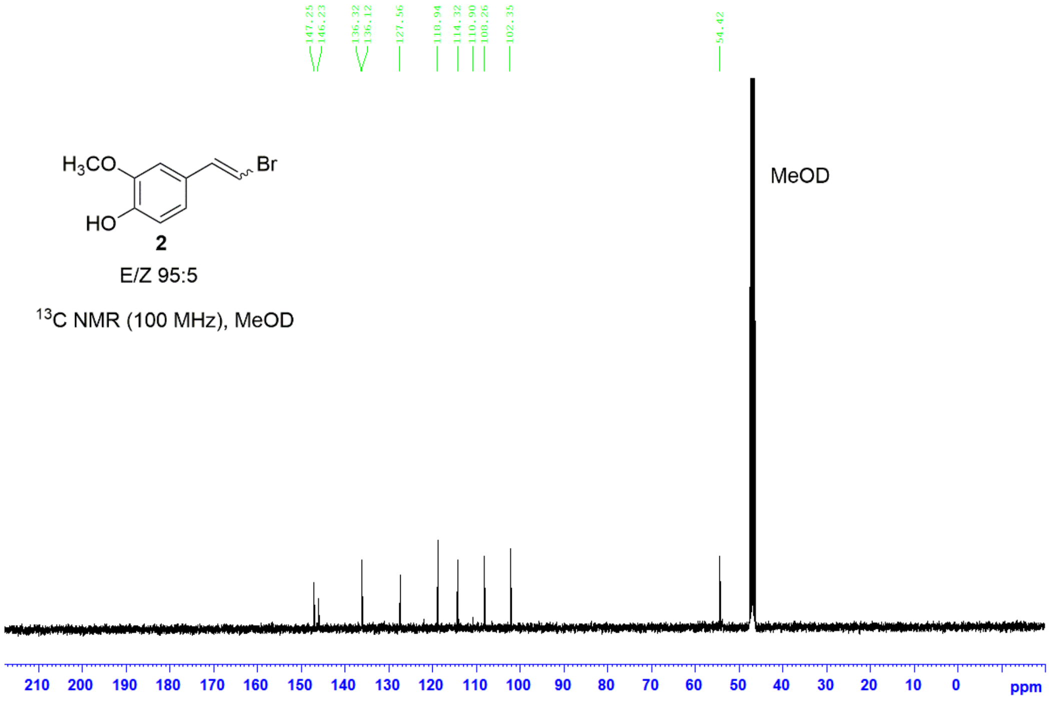

^{1}H-NMR of *E/Z* (95:5) mixture of standard product 2 with internal standard ethylene carbonate

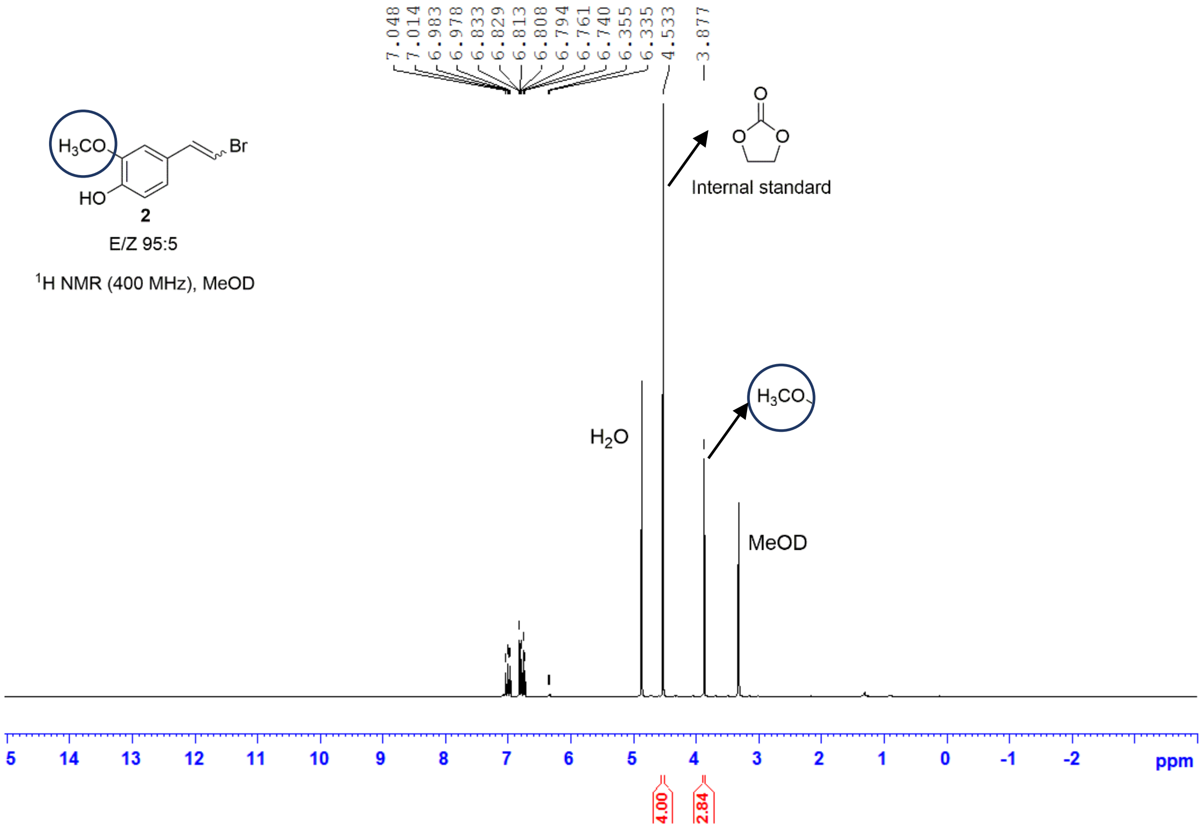

^{1}H-NMR of *E/Z* (94:6) mixture of standard product 5

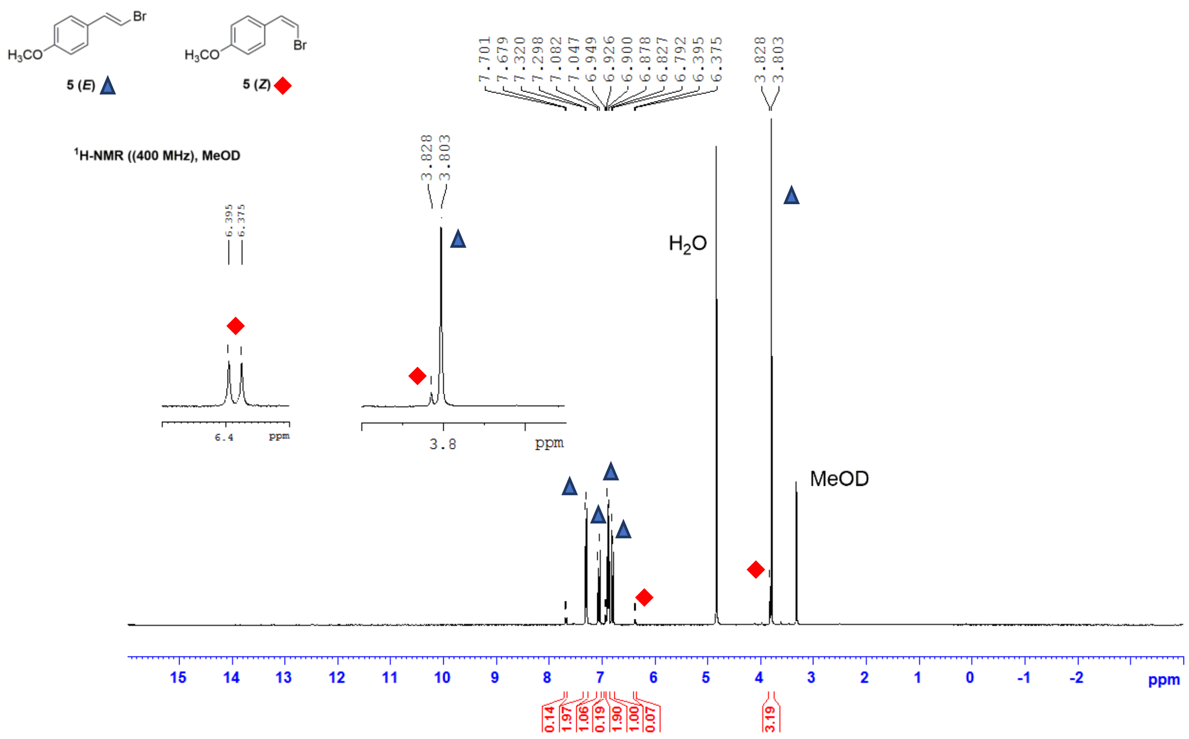

^{13}C-NMR of E/Z (94:6) mixture of standard product 5

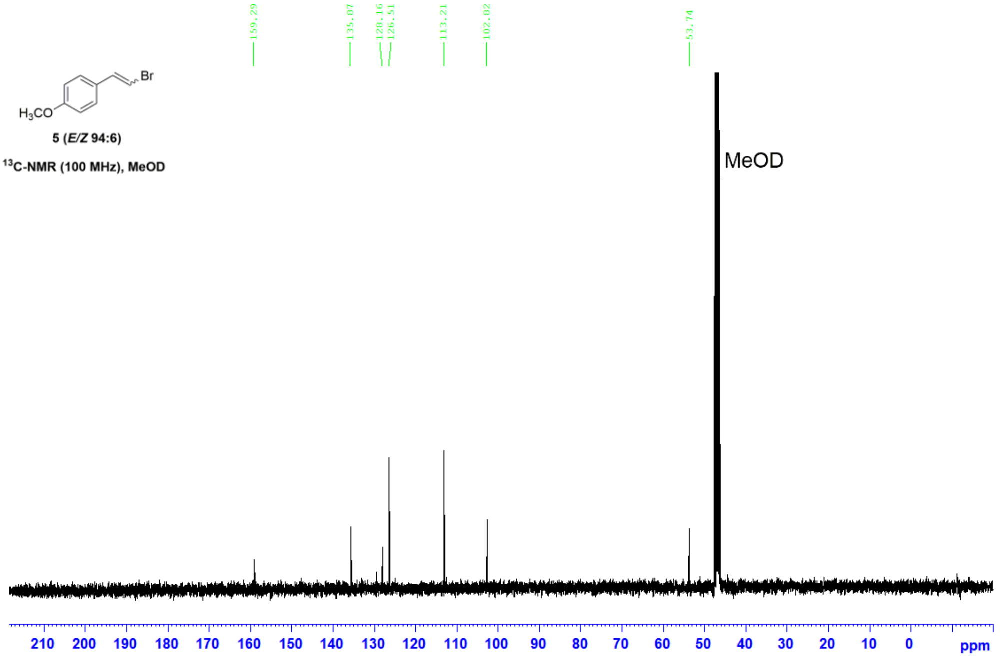

^{1}H-NMR of *E/Z* (91:9) mixture of standard product 6

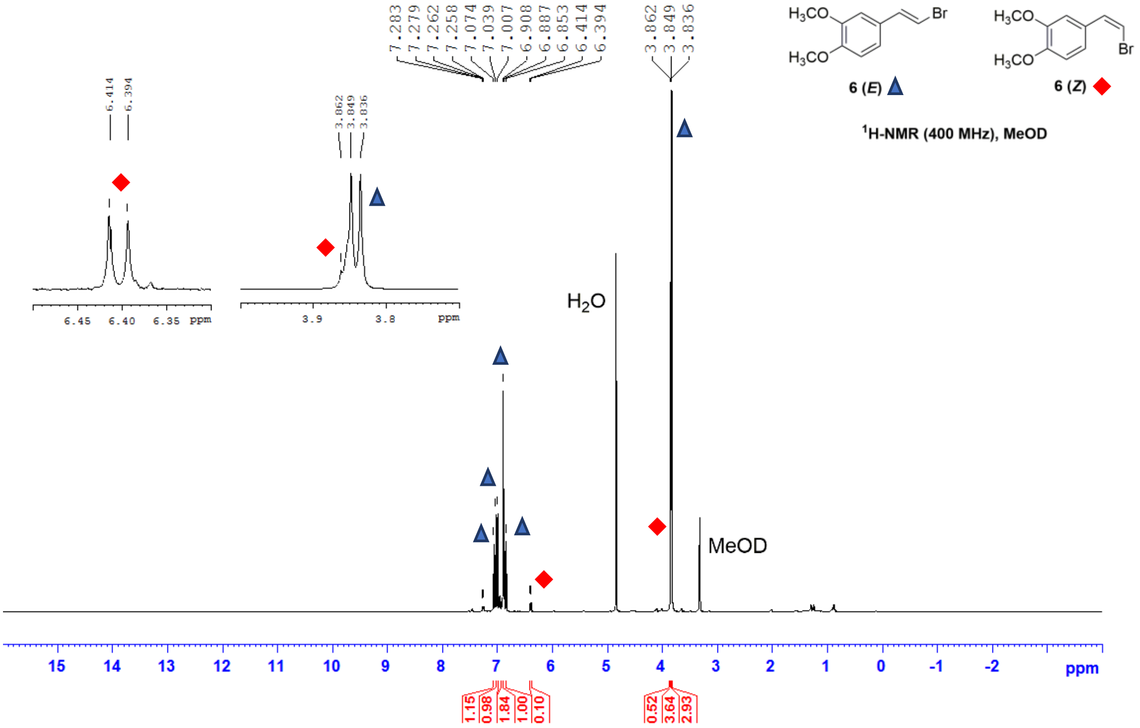

^{13}C-NMR of *E/Z* (91:9) mixture of standard product 6

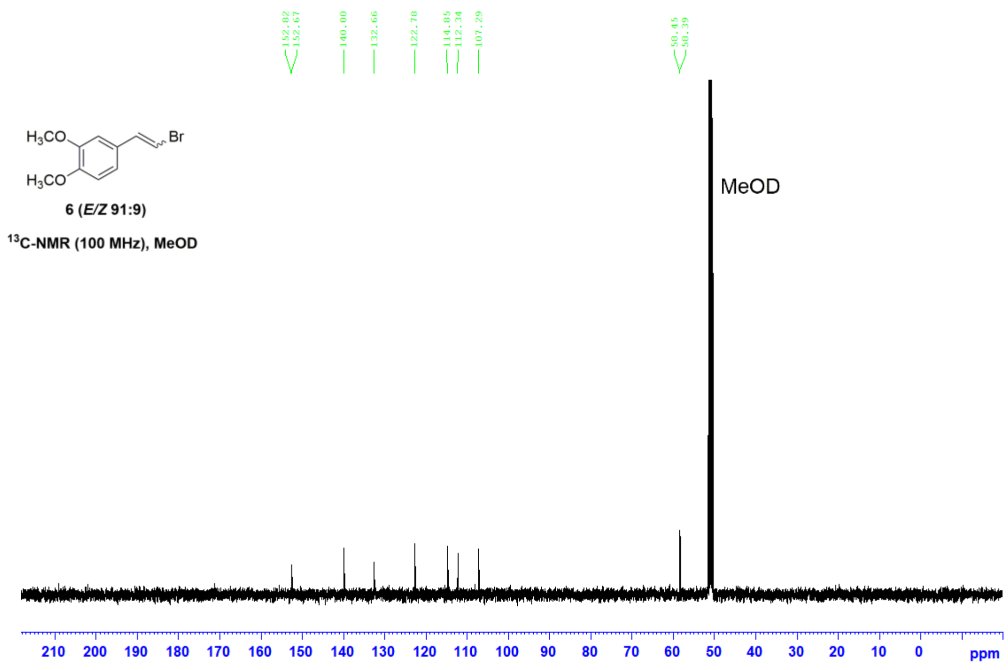

^{1}H-NMR of *E/Z* (98:2) mixture of standard product 7

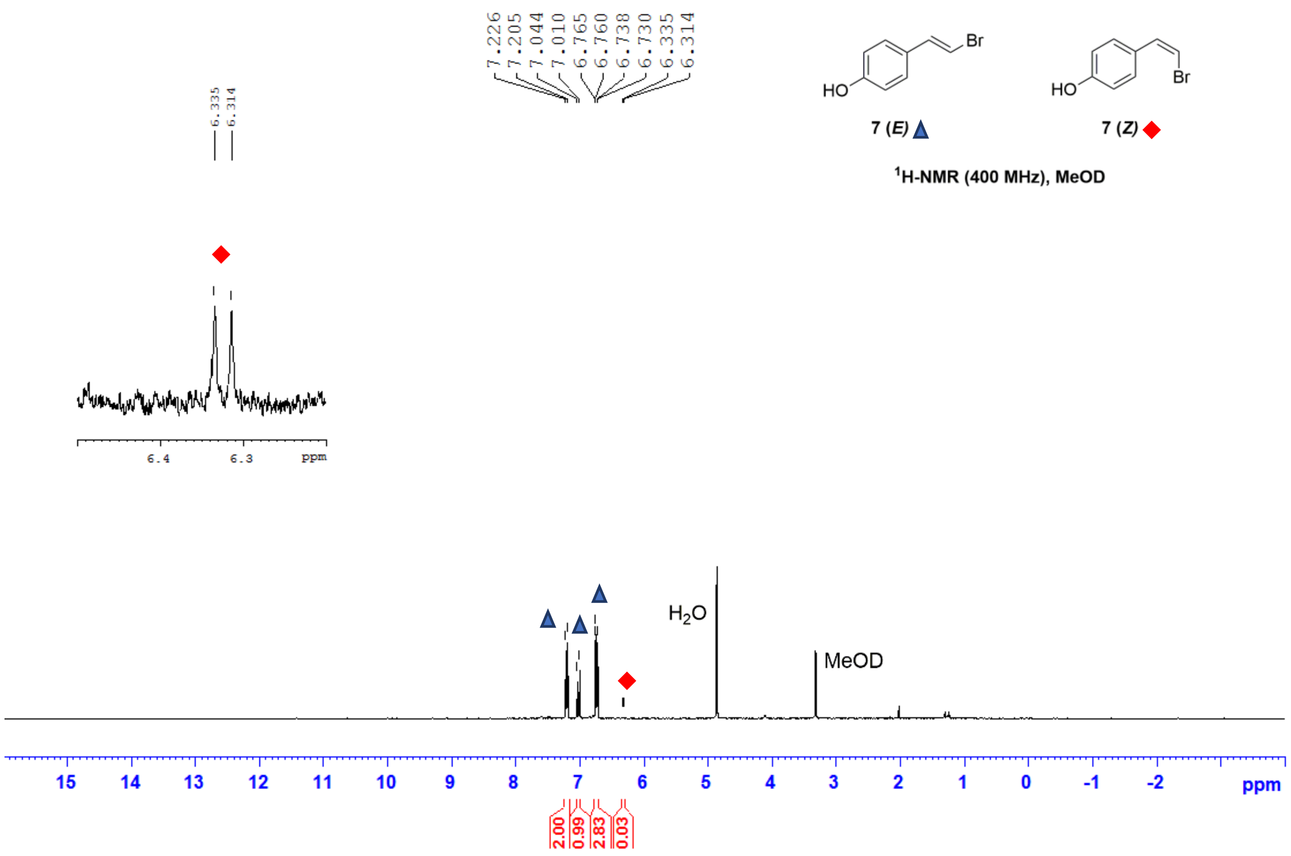

^{13}C-NMR of *E/Z* (98:2) mixture of standard product 7

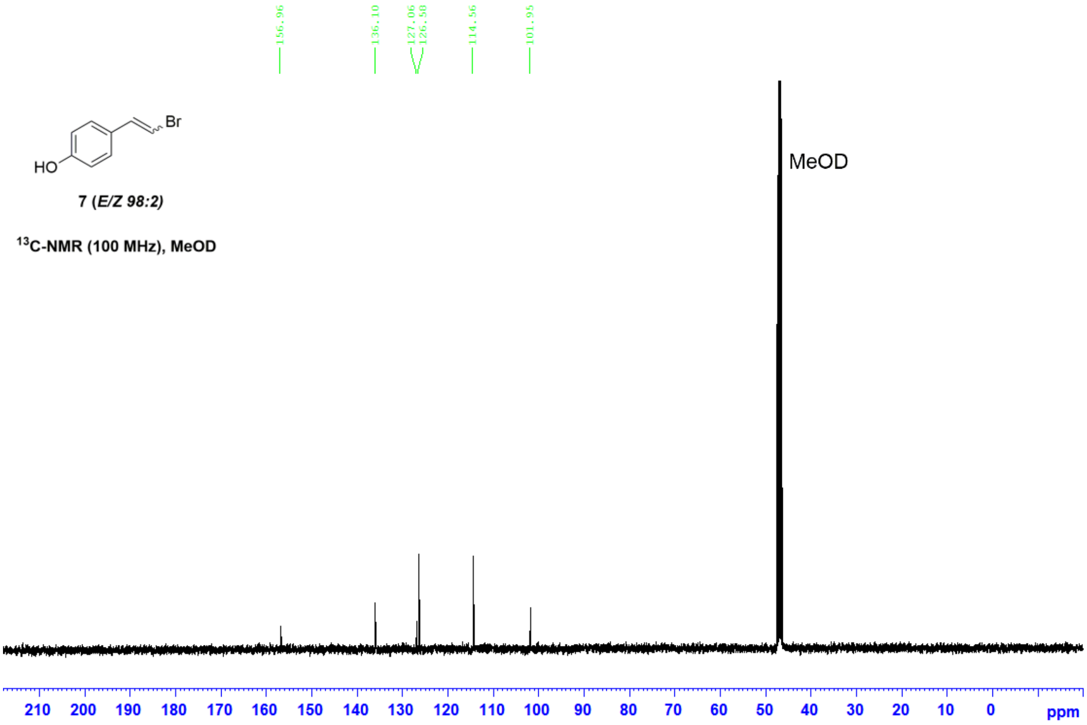

5. Determination of the hydroperoxide 4 concentration

The concentration of hydroperoxide **4** during reaction time under our reaction conditions was measured by following the oxidation of pyrogallol by horseradish peroxidase (HRP). All samples were composed by 500 μL of 2-Me-THF containing the indicated amount of *meso*-TPP, and 500 μL of citric acid buffer pH 5.0, 0.1 M. The resulted biphasic samples were stirred at room temperature, at 200 rpm in 4.0 mL glass vials placed in a jacketed beaker with commercial blue-LEDs (24W) wrapped around. At the indicated time point, 100 μL of the aqueous phase were withdrawn and added to a quartz cuvette containing a solution of 14 mM potassium phosphate 0.027% (v/v), 0.5% (w/v) pyrogallol, and 0.50 U HRP at 30 °C, and the absorbance at 420 nm was immediately measured using an Agilent Technologies Cary 60 UV-Vis spectrophotometer (equipped with a single Peltier accessory). All time-points were determined by independent duplicates and expressed as average. The obtained abs/min at 420 nm can be connected to the amount of hydroperoxide **4** contained in 100 μL of withdrawn sample by plotting it to the following calibration curve, obtained through the same pyrogallol-HRP assay at different hydrogen peroxide (H_2O_2) amounts.

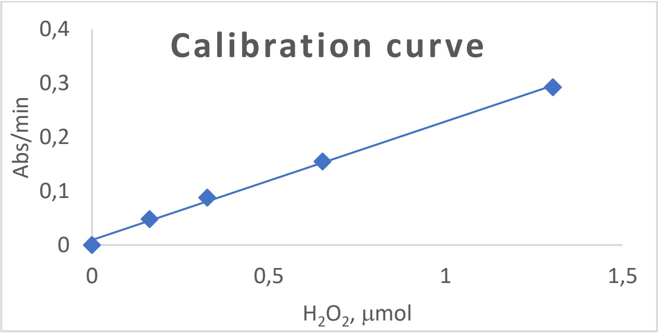

Figure S2. Calibration curve obtained at different hydrogen peroxide (H_2O_2) amounts

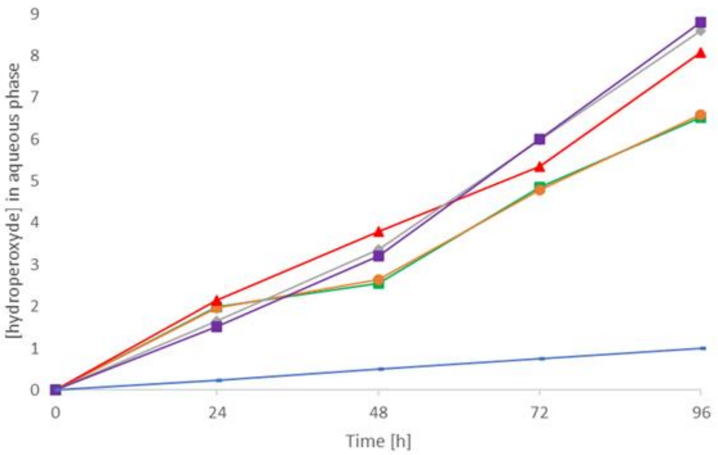

Figure S3. Time course of the concentration of the hydroperoxide **4** in the aqueous layer at different molecular oxygen and [*meso*-TPP]. [*meso*-TPP] = 400 µM (orange), 400 µM with addition of molecular oxygen (green), 200 µM (red), 100 µM (gray), 50 µM (purple), 0 µM (control, blue). All points were measured by independent duplicates and expressed as average.

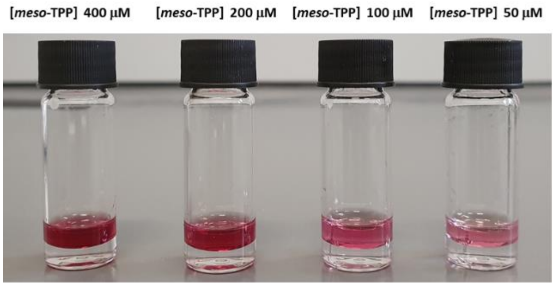

Figure S4. Appearance of the organic phase at different *meso*-TPP concentrations

6. Reaction conditions for the photochemoenzymatic Hunsdiecker-Borodin type reaction

All reactions were performed in a biphasic system composed by 500 μL of 2-methyltetrahydrofuran containing the indicated amount of α,β-unsaturated carboxylic acid and *meso*-TPP, and 500 μL of citrate buffer pH 5.0, 0.1 M, containing the indicated amount of enzyme and halide source, in 4.0 mL glass vials. The reaction was gently stirred at 200 rpm at room temperature for the indicated time in a jacketed beaker with commercial blue-LEDs (24W) wrapped around. Reactions were stopped by adding 1.0 mL of ethyl acetate. The organic layer was then separated from the aqueous one and the latter extracted four times with ethyl acetate (4x 1.0 mL). The organic layers were combined, and the solvent evaporated under vacuum. The obtained crude mixture was redissolved in 1.0 mL of acetonitrile, centrifuged, and analyzed by HPLC. Conversions and yields are always based on calibration curves with an authentic substrate standard and with chemically synthetized product standards **2**, **5-7**

6.1 HPLC chromatograms of experiments in Table 1.

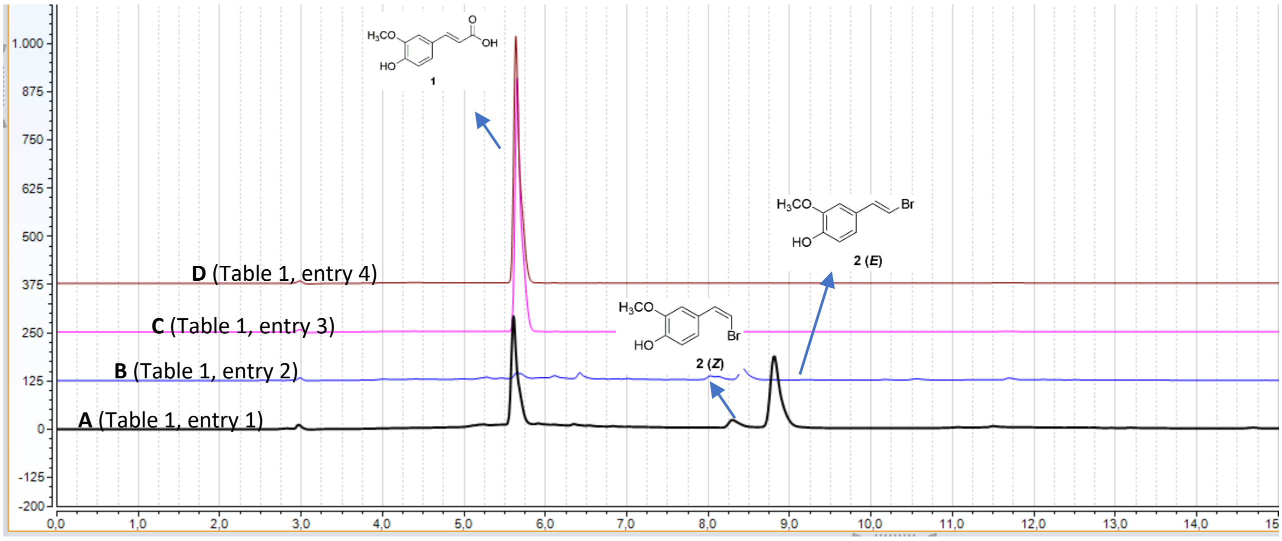

Figure S5. Reaction conditions. 2.0 mM ferulic acid and 400 μM meso-TPP in 500 μL of 2-MeTHF; 1.0 μM *Ci*VCPO and 2.0 mM KBr in 500 μL of citrate buffer pH 5.0, 0.1 M for 72 hrs (**A**); in absence of KBr (**B**); in absence of *Ci*VCPO (**C**); with thermally inactivated enzyme (**D**).

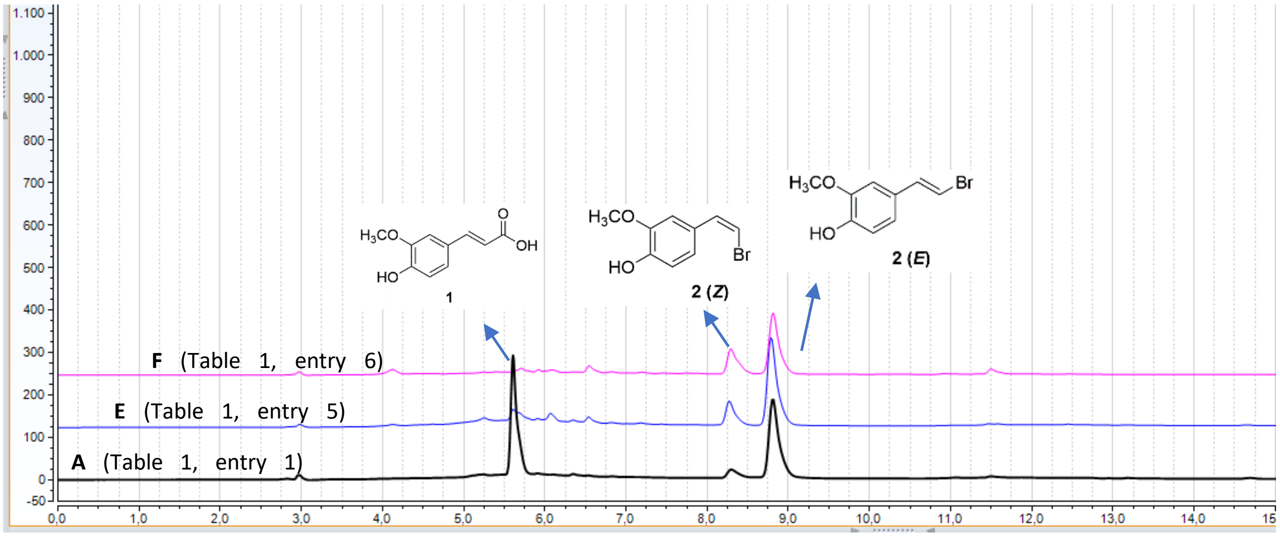

Figure S6. Reaction conditions: 2.0 mM ferulic acid and 400 μM meso-TPP in 500 μL of 2-MeTHF; 1.0 μM *Ci*VCPO and 2.0 mM KBr in 500 μL of citrate buffer pH 5.0, 0.1 M for 72 (**A**), 96 (**E**) and 120 (**F**) hours.

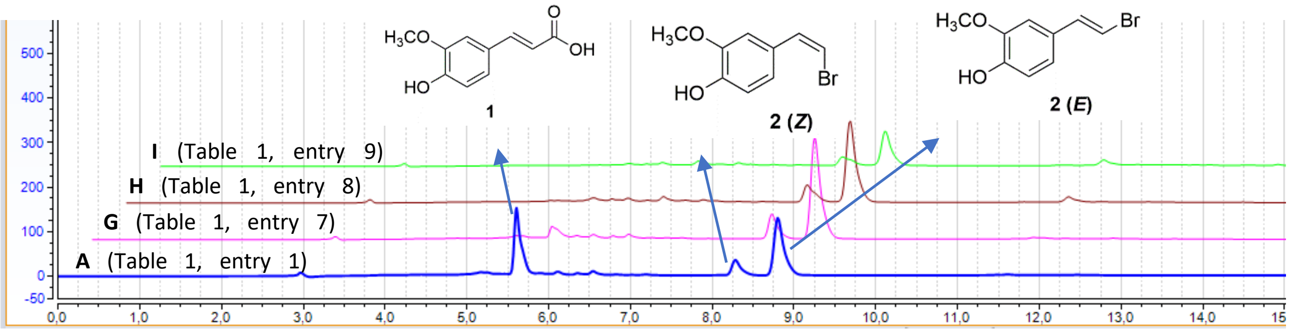

Figure S7. Reaction conditions: 2.0 mM ferulic acid and 400 μM meso-TPP in 500 μL of 2-MeTHF; 1.0 μM *Ci*VCPO and (**A**) 2.0, (**G**) 3.0, (**H**) 4.0, and (**I**) 5.0 mM KBr in 500 μL of citrate buffer pH 5.0, 0.1 M for 96 hrs.

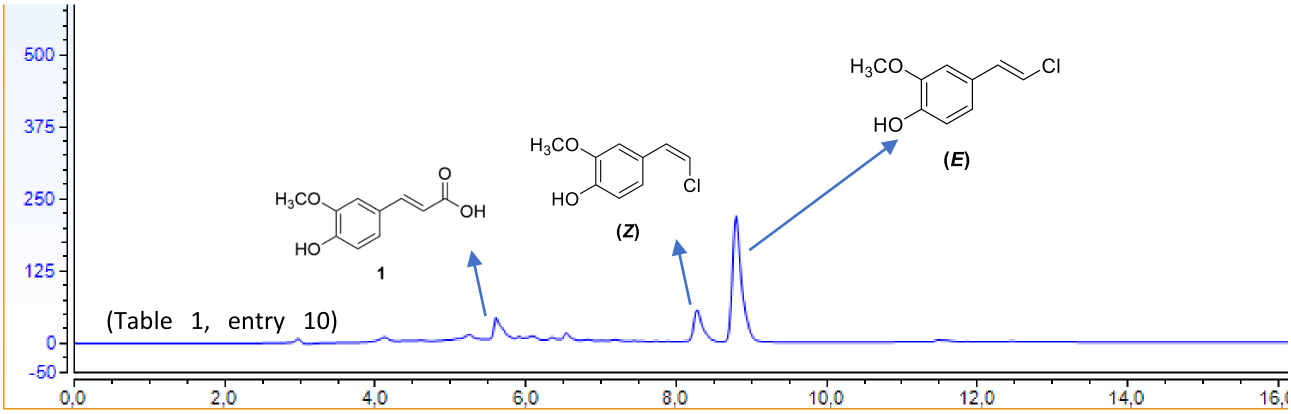

Figure S8. Reaction conditions: 2.0 mM ferulic acid and 400 μM meso-TPP in 500 μL of 2-MeTHF; 1.0 μM *Ci*VCPO and 3.0 mM KCl in 500 μL of citrate buffer pH 5.0, 0.1 M for 96 hrs.

6.2 HPLC chromatograms of experiments in Table 2

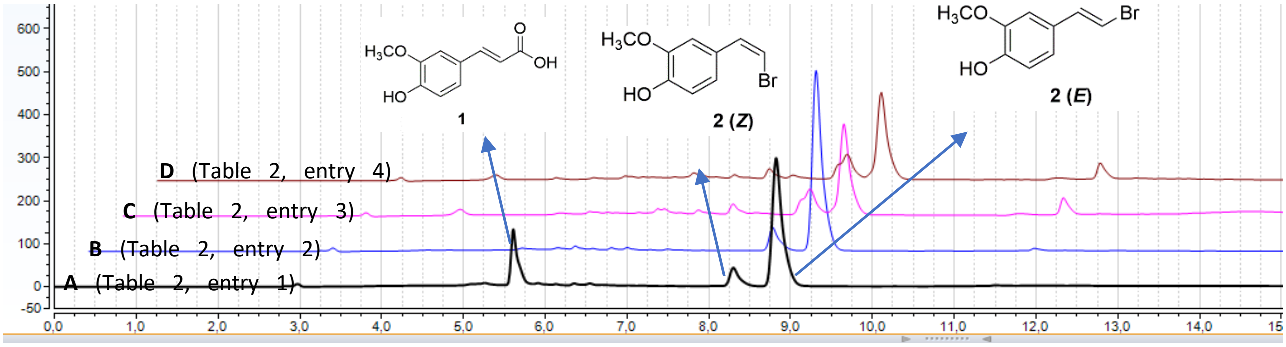

Figure S9. Reaction conditions. 2.0 mM ferulic acid and 50 μM *meso*-TPP in 500 μL of 2-MeTHF; 1.0 μM *Ci*VCPO and TBAB (**A**) 3.0, (**B**) 4.0, (**C**) 5.0 and (**D**) 6.0 mM in 500 μL of citrate buffer pH 5.0, 0.1 M for 48 hours.

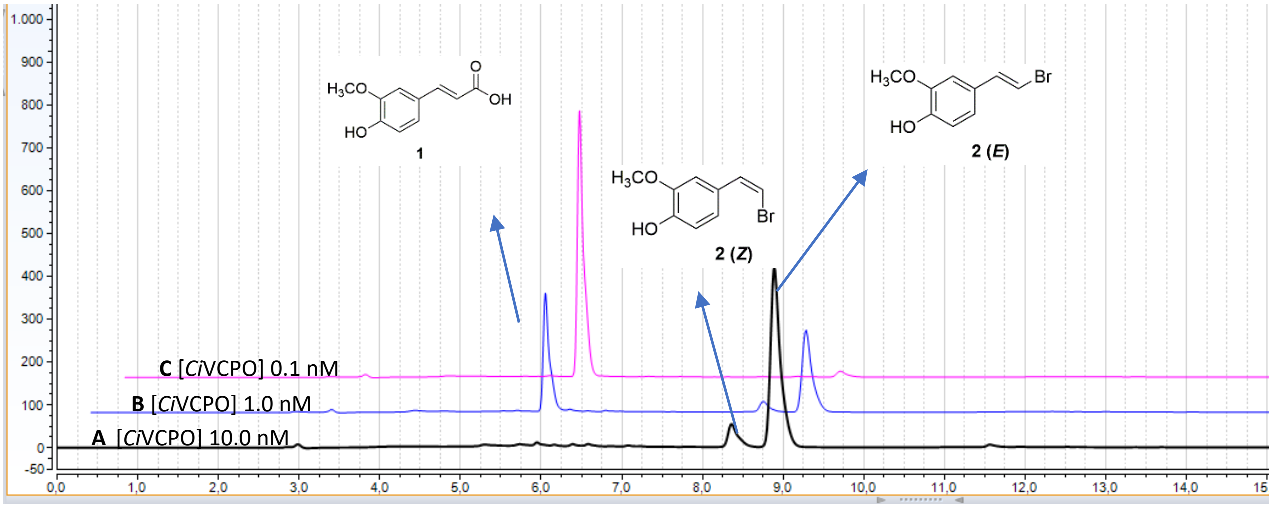

Figure S10. Reaction conditions. 2.0 mM ferulic acid and 50 μM *meso*-TPP in 500 μL of 2-MeTHF; 3.0 mM TBAB and *Ci*VCPO (**A**) 10.0, (**B**) 1.0 and (**C**) 0.1 nM in 500 μL of citrate buffer pH 5.0, 0.1 M for 48 hours.

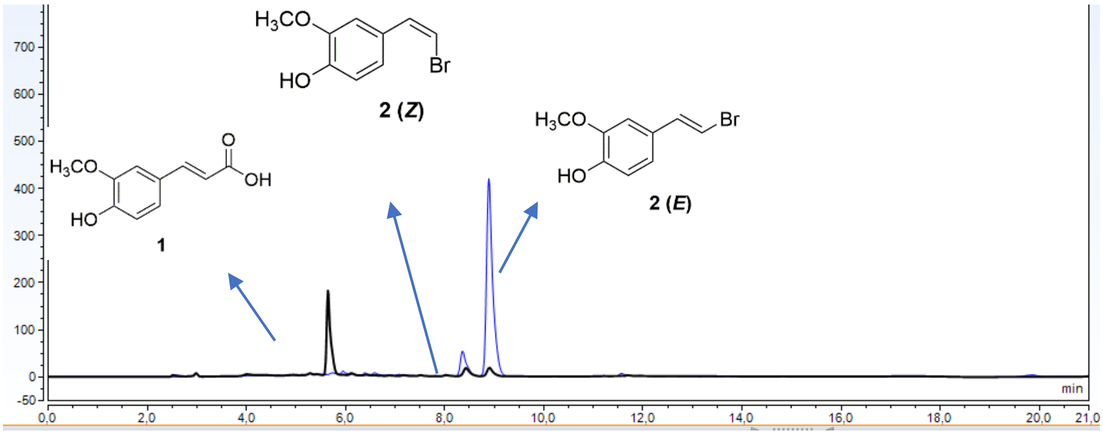

Figure S11: Comparative HPLC of reaction with KBr (**Black line**) and with TBAB (**Blue line**) after 48 h. Please note that the HPLC sample of the reaction with KBr was obtained by redissolving the crude in 2.0 mL of acetonitrile instead of 1.0 mL.

*6.3 Comparison of chemically and photochemoenzymatically obtained product **2** and HPLC purity grade*

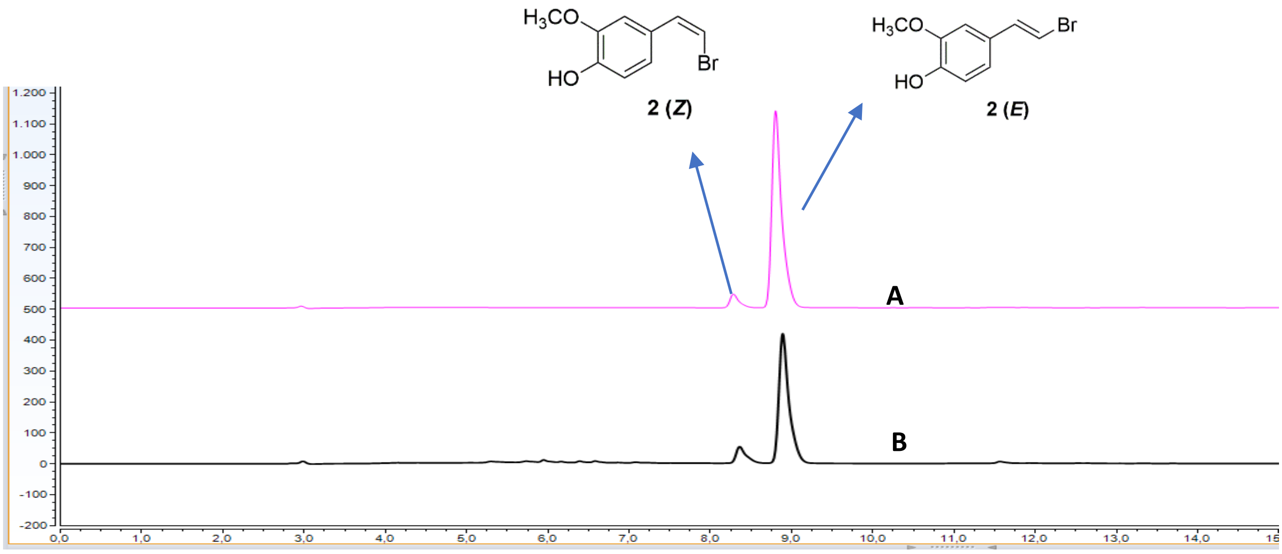

Figure S12. Comparative HPLC of (**A**) chemical standard and (**B**) our optimal reaction conditions.

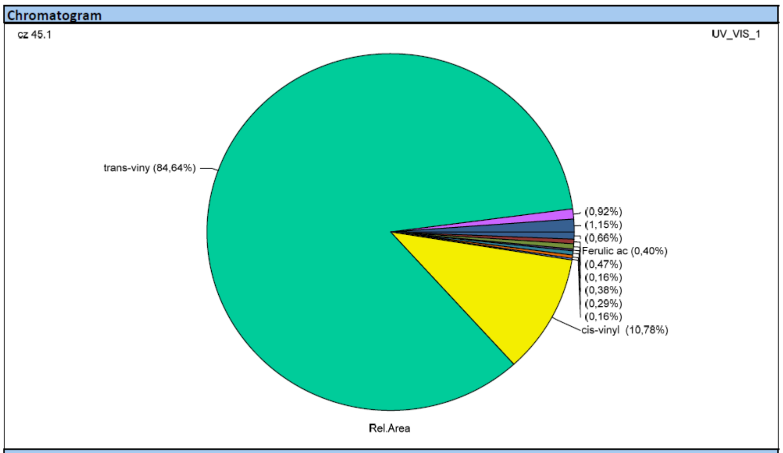

Integration Results

No.	Peak Name	Retention Time min	Area mAU*min	Height mAU	Relative Area %	Relative Height %	Amount perc %
2	Ferulic acid	5,737	0,284	3,005	0,40	0,59	0,5007
8	cis-vinyl bromide	8,360	7,647	53,904	10,78	10,65	10,6105
9	trans-vinyl bromide	8,890	60,043	418,756	84,64	82,77	60,4702
Total:			67,974	475,665	95,82	94,02	

Figure S13. HPLC purity grade of E/Z mixture of **2** obtained under optimal reaction conditions

*6.4 Comparison of chemically and photochemoenzymatically obtained product **5**,**6** and **7***

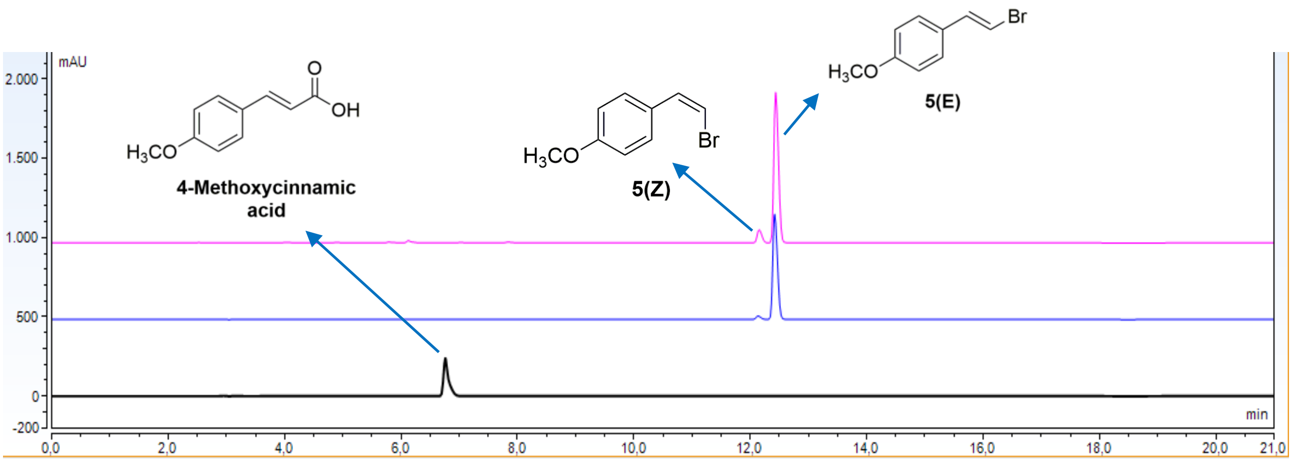

Figure S14. Comparative HPLC of substrate 4-methoxycinnamic acid (**black line**), the chemically obtained product **5** (**Blue line**) and photochemoenzymatic reaction (**pink line**). Reaction conditions: 2.0 mM 4-methoxycinnamic acid and 50 µM *meso*-TPP in 500 µL of 2-MeTHF; 4.0 mM TBAB and *Ci*VCPO 10.0 nM in 500 µL of citrate buffer pH 5.0, 0.1 M for 48 hours.

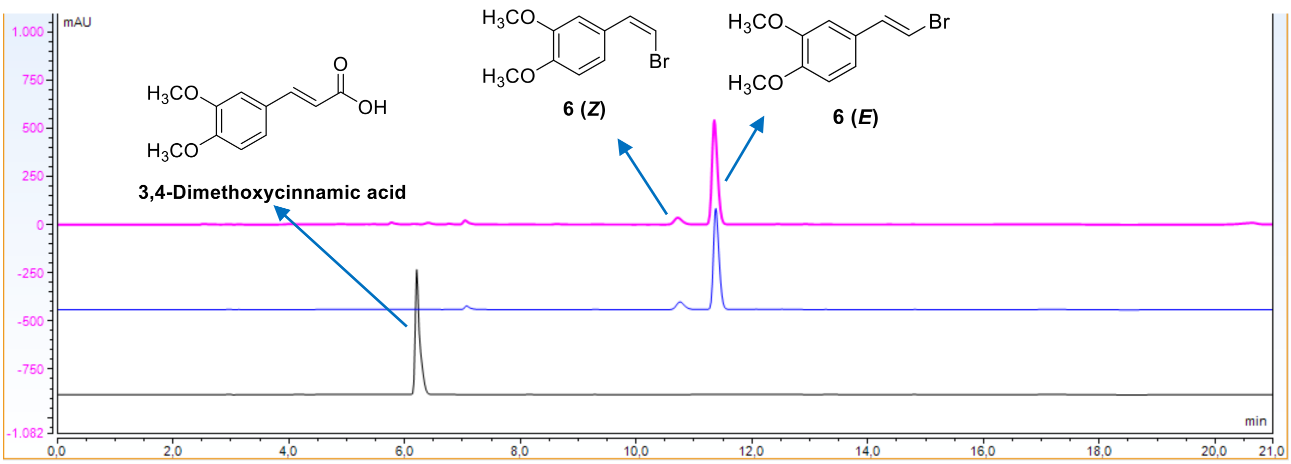

Figure S15. Comparative HPLC of substrate 3,4-dimethoxycinnamic acid (**black line**), the chemically obtained product **6** (**Blue line**) and photochemoenzymatic reaction (**pink line**). Reaction conditions: 2.0 mM 3,4-dimethoxycinnamic acid and 50 µM *meso*-TPP in 500 µL of 2-MeTHF; 4.0 mM TBAB and *Ci*VCPO 10.0 nM in 500 µL of citrate buffer pH 5.0, 0.1 M for 48 hours.

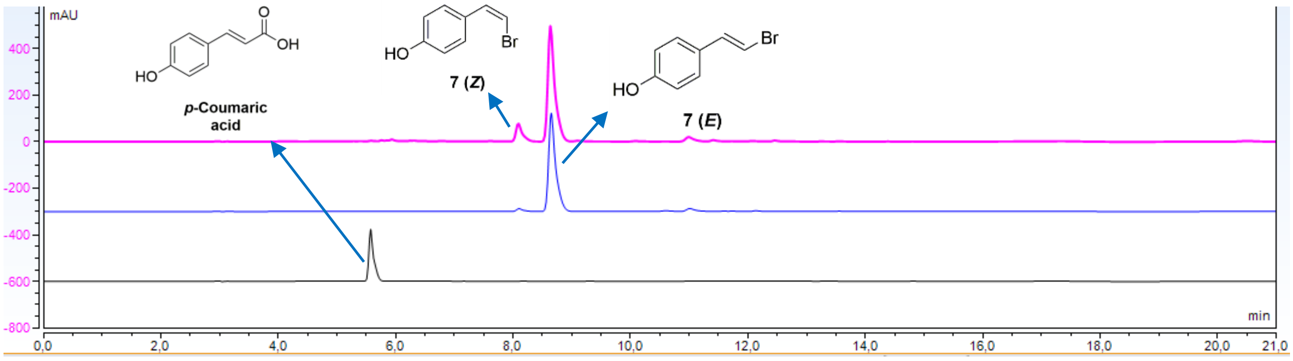

Figure S16. Comparative HPLC of substrate *p*-coumaric acid (**black line**), the chemically obtained product **7** (**Blue line**) and photochemoenzymatic reaction (**pink line**). Reaction conditions: 2.0 mM *p*-coumaric acid and 50 μM *meso*-TPP in 500 μL of 2-MeTHF; 4.0 mM TBAB and *Ci*VCPO 10.0 nM in 500 μL of citrate buffer pH 5.0, 0.1 M for 48 hours

7. Partition coefficient of ferulic acid in biphasic system under different TBAB concentrations.

All reactions were performed in a biphasic system composed by 500 μL of 2-Methyltetrahydrofuran and 500 μL of citrate buffer pH 5.0, 0.1 M, at 200 rpm in 4.0 mL glass vials, at room temperature. Vials were placed in a jacketed beaker with commercial blue-LEDs (24W) wrapped around. At indicated time points, 100 μL of the organic layer were withdrawn, and the solvent evaporated under reduced pressure. The crude residue was redissolved in 1.0 mL of acetonitrile and analyzed by HPLC. All measurements have been performed at least as duplicates from independent experiments. The concentrations of ferulic acid **1** in the organic layer were determined by calibration curves obtained with authentic standard of **1**.

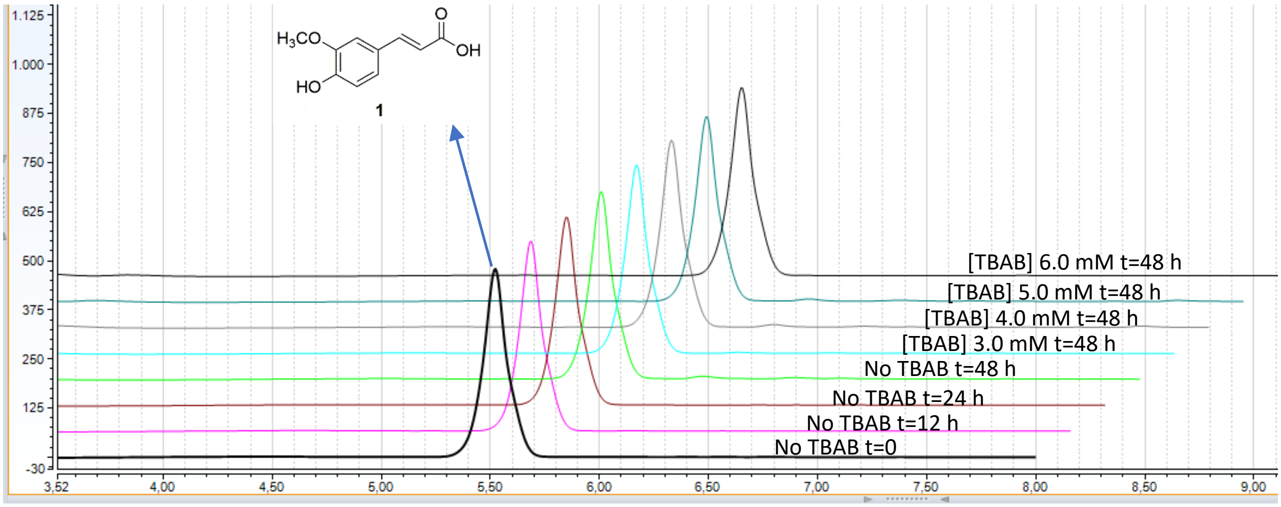

Figure S17. HPLC analysis for the measurement of the partition coefficient of ferulic acid in the biphasic system at different intervals and TBAB concentrations

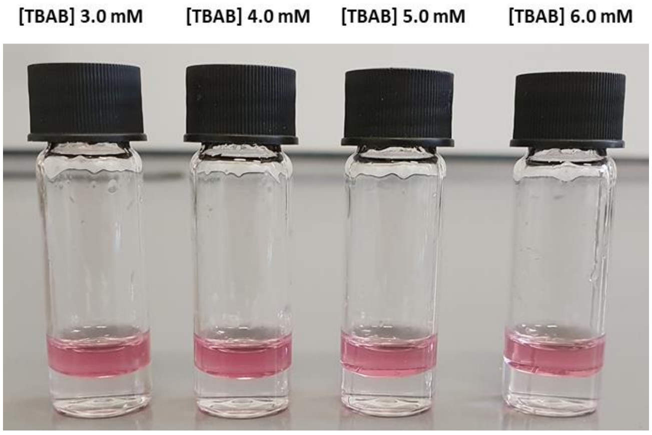

Figure S18. Appearance of the biphasic system at different TBAB concentrations after stirring it for 48 hours

8. ^{1}H-NMR of product 2 isolated from the semi-preparative photochemoenzymatic reaction.

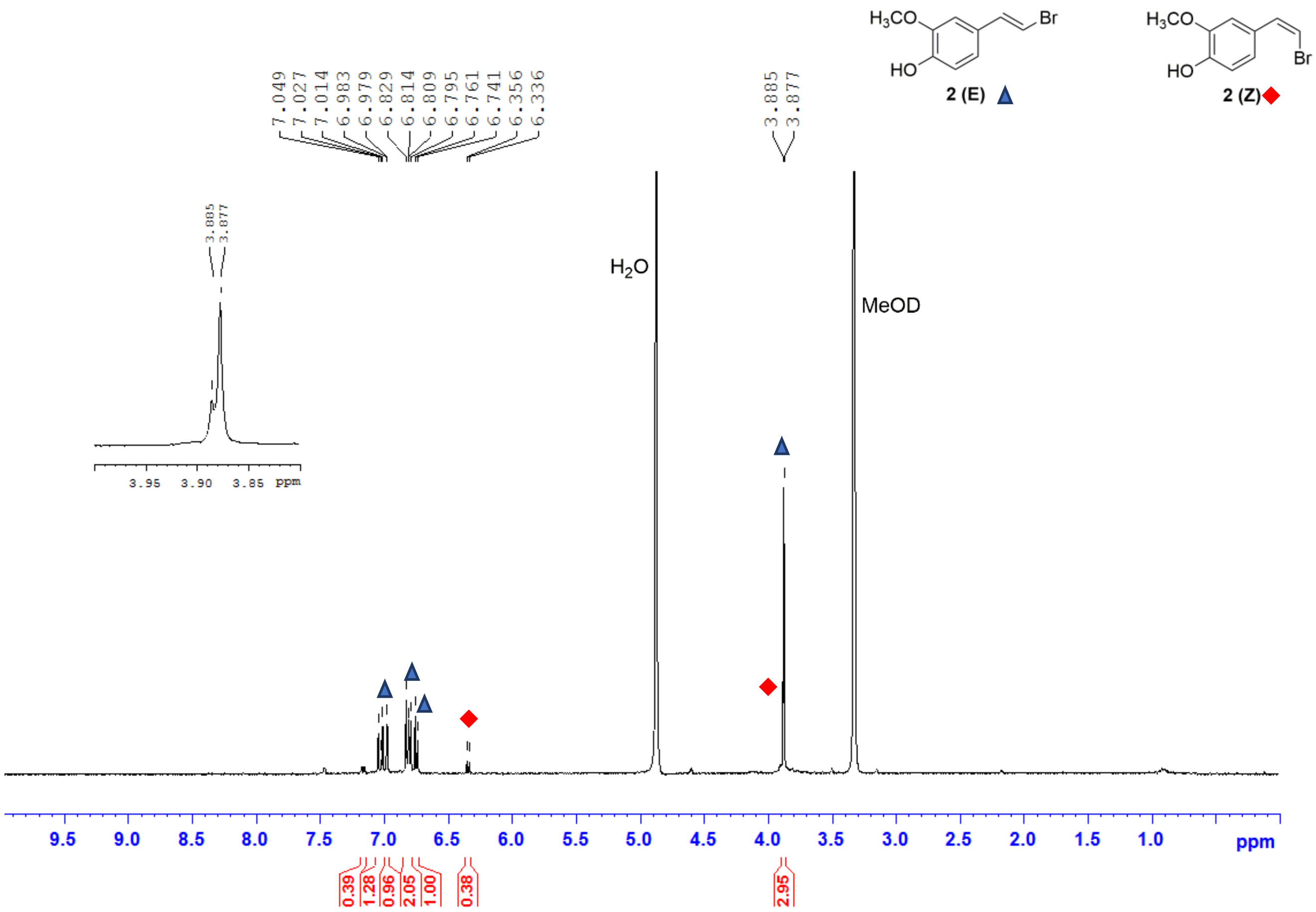

References

1. G. T. Hofler, A. But, S. H. H. Younes, R. Wever, C. E. Paul, I. Arends and F. Hollmann, *ACS Sus. Chem. Eng.*, **2020**, 8, 2602-2607.
